# Clinical characteristics and prognostic factors of COVID-19 in rheumatic patients and their family members: a retrospective study

**DOI:** 10.3389/fimmu.2024.1439242

**Published:** 2024-12-16

**Authors:** Yihua Fan, Yiwen Wang, Juanli Du, Rui Wu, Jianbin Li, Changhong Xiao, Qing Li, Mi Zhou, Ying Liu, Di Zhang, Bei Wang, Songwei Li, Zhina Zhao, Xinliang Lyu, Yuanhao Wu, Yan Liu, Xiaomei Ning, Zhiteng Li, Shujiao Yu, Ensheng Chen, Guangzhao Zhu, Yuxing Zhao, Juan Liu, Yuquan Liu, Dongyi He, Wei Liu

**Affiliations:** ^1^ Department of Rheumatism and Immunity, First Teaching Hospital of Tianjin University of Traditional Chinese Medicine, Tianjin, China; ^2^ National Clinical Research Center for Chinese Medicine Acupuncture and Moxibustion, Tianjin, China; ^3^ Department of Rheumatism and Immunity, Hospital of Chengdu University of Traditional Chinese Medicine, Chengdu, Sichuan, China; ^4^ Department of Rheumatology and Immunology, Xi’an No.5 Hospital, Xi’an, Shaanxi, China; ^5^ Department of Immunology and Rheumatology, First Affiliated Hospital of Nanchang University, Nanchang, Jiangxi, China; ^6^ Department of Rheumatology, Southern Medical University Hospital of Integrated Traditional Chinese and Western Medicine, Southern Medical University, Guangzhou, Guangdong, China; ^7^ Rheumatology Department, Qinghai Provincial Hospital of Traditional Chinese Medicine, Xining, Qinghai, China; ^8^ Department of Rheumatology, Renji Hospital, School of Medicine, Shanghai Jiao Tong University, Shanghai, China; ^9^ Department of Rheumatology, Affiliated Hospital of Shandong University of Traditional Chinese Medicine, Jinan, Shandong, China; ^10^ Department of Rheumatology and Immunology, Beijing Hospital of Traditional Chinese Medicine, Capital Medical University, Beijing, China; ^11^ Department of Rheumatism and Immunity, Henan Provincial Hospital of Chinese Medicine, Zhengzhou, Henan, China; ^12^ Department of Rheumatism and Immunity, The First Affiliated Hospital of Henan University of Chinese Medicine, Zhengzhou, Henan, China; ^13^ Rheumatology Department, Inner Mongolia Hospital of Traditional Chinese Medicine, Hohhot, Inner Mongolia Autonomous Region, China; ^14^ Department of Rheumatology, Shanghai Guanghua Hospital, Shanghai University of Traditional Chinese Medicine, Shanghai, China

**Keywords:** corona virus disease 2019, rheumatism, clinical characteristic, retrospective investigation, clinical study

## Abstract

**Background:**

Patients with rheumatic diseases who receive long-term treatment with steroids, immunosuppressants, or biologics are more susceptible to infection with pathogens than the general population. In order to explore the differences in clinical features and prognosis of Corona Virus Disease 2019 (COVID-19) infection between patients with rheumatic diseases and the general population (family members), a retrospective investigative study was used to analyze the differences between the two populations.

**Methods:**

The study was conducted in 13 Grade A Tertiary hospitals in China to investigate the clinical symptoms and prognostic factors of patients with rheumatic diseases who were infected with COVID-19 for the first time and their families.

**Results:**

A total of 2,889 participants were included in this study, including 1,530 patients with rheumatic diseases and 1,359 family members. In terms of clinical symptoms, the complete recovery time from COVID-19 for patients with rheumatic disease patients was 13 days (8.00, 18.00), which was shorter than that of family members (16 days, 11.00, 20.00). The risk of developing moderate to severe cases of COVID-19 was lower in patients with rheumatic disease than in their family members (OR=0.511, *P*=0.0026). Compared with non-use of non-steroidal anti-inflammatory drugs (NSAIDs), the risk of developing mild cases of COVID-19 was 0.595 times greater with pre-infection use of NSAIDs (*P* = 0.0003). The use of glucocorticoids and Chinese herbal decoctions before infection increased the probability of developing mild cases of COVID-19 (OR=1.537, 1.773, *P*<0.05). The risk of developing moderate to severe cases with disease-modifying anti-rheumatic drugs (DMARDs) used before infection was 0.350 times that without such drugs (*P*<0.001). In terms of prognosis, compared with family members, the complete recovery time of patients with rheumatic diseases was reduced by 2.241 days on average (*P*<0.001), and the complete recovery time of patients with mild rheumatism was reduced by 4.178 days on average (*P*<0.001). There was no significant difference in the complete recovery time from COVID-19 in patients with severe rheumatism compared with their family members (*P*=0.1672). The use of NSAIDs, glucocorticoids, DMARDs, biologics, Chinese patent medicine, and Chinese herbal decoctions during the infection period could shorten the recovery time of COVID-19 symptoms (*P*<0.05).

**Conclusions:**

Compared with their family members, patients with rheumatic diseases had milder symptoms after infection with COVID-19, which was related to the use of glucocorticoids, DMARDs, and Chinese herbal decoctions before infection. During the COVID-19 infection phase, the use of NSAIDs, glucocorticoids, DMARDs, biologics, Chinese patent medicine, and Chinese herbal decoctions might shorten the recovery time from symptoms of COVID-19.

**Chinese clinical trial registry:**

ChiCTR2300072679

## 1 Introduction

The Corona Virus Disease 2019 (COVID-19) is an acute respiratory infectious disease caused by the SARS-CoV-2 (1), which mainly manifests with symptoms such as fever, cough, and fatigue. It is primarily transmitted through the respiratory tract, while contacting with contaminated items can also cause infection, and the population is generally susceptible (2, 3). By the end of 2022, the World Health Organization (WHO) has defined five variants of concern (VOCs), namely Alpha, Beta, Gamma, Delta and Omicron (4). According to WHO monitoring data, since February 2022, Omicron variants have accounted for more than 98%, and have become the world’s main new coronavirus epidemic strain, posing a huge challenge to global public health (5, 6). According to the European Centre for Disease Prevention and Control, from December 2019 to December 2022, the mortality rate of COVID-19 patients was 7.04% globally, and individual countries may have higher mortality rates (7). From the perspective of virus mutation, experts generally believe that the general direction of virus mutation is lower pathogenicity, and the novel coronavirus will exist in nature for a long time, whose pathogenicity is significantly reduced compared with the early stage, and the disease will gradually evolve into a common respiratory infectious disease (8, 9).

Rheumatic diseases are autoimmune disorders, and in China, the incidence of rheumatic diseases is only lower than circulatory system diseases and endocrine and metabolic diseases, ranking third (10). Due to long-term use of steroids and immunosuppressants, patients with rheumatic disease have low immune function, which increases their risk of contracting COVID-19 (11). Due to the characteristics of rheumatic diseases, many patients with rheumatic disease have some symptoms in the active period that are similar to the clinical manifestations of COVID-19, and some drugs used by them may alleviate the clinical symptoms of COVID-19 (12). Currently, it is unclear whether there are differences in clinical features and prognosis between patients with rheumatic diseases and the general population after infection with the COVID-19. Some studies have indicated that the clinical manifestations of patients with rheumatic diseases infected with the COVID-19 are similar to those of the general population (13). Therefore, there is still controversy about the differences in symptoms and prognosis between patients with rheumatic diseases and the general population after infection with COVID-19, which is not conducive to the clinical treatment of patients with rheumatic diseases and their infection with COVID-19.

Therefore, we conducted a retrospective investigative analysis to explore the differences of the clinical features and prognosis after infection with COVID-19 between patients with rheumatic diseases and their family members as the general population, to provide evidence for the clinical treatment of patients with rheumatic diseases.

## 2 Research methods

### 2.1 Research design

This study was a retrospective investigative study investigating patients with rheumatic diseases and the general population who were infected with the COVID-19 for the first time, starting from May 2023 in the rheumatology and immunology departments of 13 Grade A Tertiary hospitals in China, including First Teaching Hospital of Tianjin University of Traditional Chinese Medicine, Hospital of Chengdu University of Traditional Chinese Medicine, Xi’an No.5 Hospital, First Affiliated Hospital of Nanchang University, Southern Medical University Hospital of Integrated Traditional Chinese and Western Medicine, Qinghai Provincial Hospital of Traditional Chinese Medicine, Renji Hospital, Affiliated Hospital of Shandong University of Traditional Chinese Medicine, Beijing Hospital of Traditional Chinese Medicine, Henan Provincial Hospital of Chinese Medicine, The First Affiliated Hospital of Henan University of Chinese Medicine, Inner Mongolia Hospital of Traditional Chinese Medicine, Shanghai Guanghua Hospital. In order to ensure the two groups of patients infected with the same strain as possible, at the same time, to avoid the influence of living environment and diet on the course of the COVID-19 infection, the general population in this study was selected from the family members of patients with rheumatic disease. A questionnaire survey was conducted on patients with COVID-19 who met the criteria. Physicians in the rheumatology and immunology department filled out the questionnaire independently, and statistical personnel conducted statistical analysis after collecting clinical data of patients. This study was approved by the Ethics Committee of First Teaching Hospital of Tianjin University of Traditional Chinese Medicine (TYLL2023(Z)010). It was also registered in the Chinese Clinical Trial Registry (ChiCTR2300072679).

### 2.2 Research population

1. Enrolled population: Individuals with rheumatic diseases and their family members who were infected with the COVID-19 for the first time.
2. Diagnostic criteria: ①Diagnosis of COVID-19 referred to the *Diagnosis and Treatment of COVID-19 (10th edition)* published by the National Health Commission of the People’s Republic of China (3). ②Rheumatic diseases included systemic lupus erythematosus, rheumatoid arthritis, ankylosing spondylitis, Sjogren’s syndrome, etc. (14), and the diagnostic criteria referred to the diagnostic criteria issued by the American College of Rheumatology.
3. Inclusion criteria: ①Patients with rheumatic diseases who met any one or more of the diagnostic criteria for rheumatism and had been taking one or more immunosuppressants, such as glucocorticoids, disease-modifying anti-rheumatic drugs (DMARDs), biologics, for at least 1 month before infection with COVID-19;②Patients met the diagnostic criteria for COVID-19 infection, and it was the first infection; ③Aged ≥18 years old; ④The patient had clear consciousness and could complete the questionnaire independently; ⑤Patients agreed to participate in this study and signed informed consent.
4. Exclusion criteria: Patients who met any of the following criteria would not be allowed to participate in this study: ①Patients with other respiratory diseases that affected the observation of the disease except for the COVID-19 infection; ②Women who were pregnant, planning to become pregnant or breastfeeding; ③Patients with mental disorders, severe cognitive dysfunction, language impairment, and difficulty in obtaining accurate disease data.

### 2.3 Exposure factors

The exposure factor in this study was whether the patient had rheumatic disease. Based on whether the patient experienced rheumatic disease, the patients were divided into two groups: those with rheumatic disease who had been infected with the COVID-19 and those without rheumatic disease who had been infected with the COVID-19. In order to ensure the two groups of patients have the same virus infection, and also to avoid the influence of living environment and diet on the course of the COVID-19 infection, the general population in this study was selected from the family members of patients with rheumatic disease.

### 2.4 Outcome measures

#### 2.4.1 Primary outcome measures

Complete recovery time from COVID-19: The time from the date of diagnosis of the patient to the negative nucleic acid test (and complete recovery of symptoms). In order to explore whether the condition of rheumatic disease affected the complete recovery time from COVID-19, we divided the patients with rheumatic disease into two subgroups (classified according to the degree of rheumatic disease activity): mild and severe. According to the recommendations of the American College of Rheumatology or the European League of Rheumatology, rheumatoid arthritis was graded according to Disease Activity Score 28 (DAS28) (15), in which a DAS28 score ≤ 2.6 was considered as clinical remission, and 2.6 < DAS28 score ≤ 3.2 was considered as low disease activity. These two conditions were called mild rheumatism in this study, otherwise it was severe rheumatism. Systemic lupus erythematosus disease activity index (SLEDAI) was used to grade systemic lupus erythematosus (16), in which SLEDAI score ≤ 4 was classified as basically inactive, and SLEDAI score ≤ 5 was classified as mildly active. These two conditions were called mild rheumatism in this study, otherwise it was severe rheumatism. Ankylosing Spondylitis was graded according to the Bath Ankylosing Spondylitis Disease Activity Index (BASDAI) (17), in which BASDAI < 4 indicated inactivity, and BASDAI ≥ 4 indicated disease activity. In this study, inactivity was referred to mild rheumatism, while disease activity was referred to severe rheumatism. According to EULAR Sjogren’s syndrome disease activity index (ESSDAI), the activity level of Sjogren’s syndrome was graded (18). In the ESSDAI score, each item was classified into: inactive, mildly active, moderately active, and highly active. Among them, inactivity and mild activity were referred to mild rheumatism, while moderate activity and high activity were classified as severe rheumatism.

The clinical type of COVID-19 infection was classified into two outcomes: mild and moderate to severe.

#### 2.4.2 Secondary outcome measures

Duration of symptoms: the time (days) from the onset of symptoms to complete recovery of symptoms.

### 2.5 Sample size calculation

Multivariate regression model was used as the main analysis method in this study. Referring to previous studies (19), the number of included cases was at least 20-30 times that of the pre-selected factors. Since there were 55 pre-selected factors in this study, at least 1650 cases should be included in this study.

### 2.6 Research procedure

(1) Disease classification of patients infected with COVID-19: all eligible and ineligible patients would be classified into different severity levels by physicians before completing the questionnaire. The specific clinical classification criteria were as follows (3):

- Mild. Clinical manifestations: mainly upper respiratory infection symptoms such as dry throat, sore throat, cough, fever, etc.
- Moderate. Persistent high fever > 3 days or (and) cough, dyspnea, but respiratory rate (RR) < 30 times/min, SpO2 > 93% at rest with air breathing. Imaging showed the characteristic manifestations of COVID-19 pneumonia.
- Severe. Adults met any of the following criteria and could not be explained by other causes other than COVID-19 infection: ① Dyspnea, RR ≥ 30 times/min; ② SpO_2_ ≤ 93% at rest with air breathing; ③The arterial partial pressure of oxygen and (P_a_O_2_)/the fraction of inspired oxygen (FiO_2_) ≤ 300mmHg (1mmHg=0.133kPa); ④Clinical symptoms progressively worse, and lung imaging showed a > 50% increase in lesions within 24-48 hours.
- Critical. One of the following conditions: ① Respiratory failure, requiring mechanical ventilation; ② Shock; ③ Combined with other organ failure requiring ICU monitoring and treatment.

In addition to the above four types, there was a special case that a patient’s nucleic acid test was positive for the COVID-19, but the patient had no clinical symptoms, which was called asymptomatic type.

(2) Questionnaire survey: All surveyors involved in the survey at the center were clinical physicians specializing in rheumatic diseases. The surveyors were trained by the chief physician and qualified before conducting clinical surveys. The clinical physicians first explained the clinical significance and survey content of the questionnaire survey form to the patients, and then the surveyors inquired about the basic information and disease status of the patients and filled out the survey form honestly. The content of the survey form included the basic information of the patients (such as name, gender, age, height, weight, etc.), the basic situation of patients with rheumatic disease (such as the name and course of the disease, drug use before and after contracting COVID-19, etc.), and the conditions related to COVID-19 (such as clinical manifestations, symptom duration, drug use for treatment, complete recovery time, and duration of each symptom, etc.).

(3) Questionnaire collection: The data collection for this study would be conducted by trained surveyors at each center who had received standardized training. All data would be recorded into Excel tables after being double-checked for accuracy.

### 2.7 Statistical analysis

SPSS 21.0 software and R 4.3.0 software were used for statistical analysis. For quantitative data, the normal distribution was expressed as the mean ± standard deviation (Mean ± SD) and the skew distribution was expressed as M (P_25_, P_75_). Baseline data and duration of individual symptoms of the two groups were compared. If the measurement data were consistent with normal distribution and homogeneity of variance, t test was used; if not, non-parametric test was used. For counting data, the percentage was described, and chi-square test was used for comparison between groups.

With the complete recovery time from COVID-19 as the outcome, multiple linear regression was used to explore the effects of related factors (whether suffering from rheumatism and medication usage before infection, such as NSAIDs, DMARDs, glucocorticoids, biologics, Chinese patent medicine, Chinese herbal decoctions) on the complete recovery time from COVID-19. Confounding variables (correction factors) selected in this study included gender, age, BMI, diabetes mellitus, hypertension, heart disease, hyperlipidemia, other underlying diseases, years of rheumatism, clinical type of infection with COVID-19, and whether antiviral drugs, antibiotics, expectorants, vitamins used during infection. *P* < 0.05 was considered to be statistically significant.

With the clinical types of COVID-19 infection (asymptomatic, mild, moderate to severe) as the outcome, multiple logistic regression was used to explore the effects of related factors (whether suffering from rheumatism and medication usage before infection, such as NSAIDs, DMARDs, glucocorticoids, biologics, Chinese patent medicine, Chinese herbal decoctions) on clinical symptoms. Confounding variables (correction factors) selected in this study included: gender, age, BMI, diabetes mellitus, hypertension, heart disease, hyperlipidemia, other underlying diseases, and years of rheumatism. *P* < 0.05 was considered to be statistically significant.

## 3 Results

### 3.1 Baseline information

A total of 2889 respondents were included in this study, including 1530 patients with rheumatic diseases and 1359 family members. Among these patients with rheumatic diseases, there were 718 cases of those solely diagnosed with rheumatoid arthritis, 385 cases of those solely diagnosed with systemic lupus erythematosus, 185 cases of those solely diagnosed with ankylosing spondylitis, 104 cases of those solely diagnosed with Sjögren's syndrome, 13 cases of other rheumatic diseases, and 125 cases of patients with multiple rheumatic diseases. **Table 1** describes the baseline characteristics of patients with rheumatic diseases and their families. Among 1530 patients with rheumatic diseases, 1256 were women and 274 were men, with a median age of 46 years. Among the 1359 family members, 671 were female and 688 were male, with a median age of 44 years. Among 1530 patients with rheumatic diseases infected with COVID-19, 202 were asymptomatic, 1233 were mild, and 95 were moderate to severe. Among 1,359 family members, 215 were asymptomatic, 972 were mild and 172 were moderate to severe. There were statistical differences between the two groups in gender, age, height, weight, BMI, whether they had diabetes, hyperlipidemia, and other underlying diseases (*P* < 0.05).

**Table 1:** Baseline characteristics of 2889 patients infected with COVID-19.

Variables	Rheumatism group	Family members group	*P*
Cases (n)	1530	1359	/
Duration of rheumatism	7.0(3.0, 13.0)	/	/
Gender (n,%)			
Female	1256(82.1)	671(49.4)	<0.001
Male	274(17.1)	688(50.6%)	
Age	46.0(36.0,57.0)	44.0(30.0,56.0)	<0.001
Height (cm)	161.0(158.0,166.0)	168.0(160.0,173.0)	<0.001
Weight (kg)	59.0(53.0, 65.00)	65.0(55.0, 74.0)	<0.001
BMI	22.3(20.4, 24.7)	23.1(20.8, 25.4)	<0.001
Comorbidities (Yes), n(%)			
Underlying diseases	434(28.4)	351(25.8)	0.132
Hypertension	244(15.9)	217(16.0)	1
Diabetes	68(4.4)	85(6.3)	0.031
Heart diseases	61(4.0)	55(4.0)	1
Hyperlipidemia	87(5.7)	50(3.7)	0.014
Other underlying disease	147(9.6)	88(6.5)	0.002
Clinical classification of rheumatism (n)			
Mild	713	/	/
Severe	817	/	/
Clinical types of COVID-19 infection (n)			
Asymptomatic	202	215	<0.001
Mild	1233	972	
Moderate to severe	95	172	
Complete recovery time from COVID-19	13.0(8.0, 18.0)	16.0(11.0, 20.0)	<0.001
Use medication before infection (Yes), n(%)			
NSAIDs	401(26.2)	29(2.1)	<0.001
Glucocorticoids	475((31.0)	28(2.1)	<0.001
DMARDs	878(57.4)	0(0.0)	<0.001
Biologics	428(30.0)	0(0.0)	<0.001
Chinese patent medicine	206(13.5)	12(0.9)	<0.001
Chinese herbal decoctions	388(25.4)	20(1.5)	<0.001
Use medication during infection (Yes), n(%)			
NSAIDs	723(47.3)	739(54.4)	<0.001
Glucocorticoids	203(13.3)	46(3.4)	<0.001
DMARDs	85(5.6)	2(0.1)	<0.001
Biologics	67(4.4)	0(0.0)	<0.001
Chinese patent medicine	523(34.2)	357(26.3)	<0.001
Chinese herbal decoctions	30(2.0)	54(4.0)	<0.001

### 3.2 Clinical symptoms

**Figure 1:**
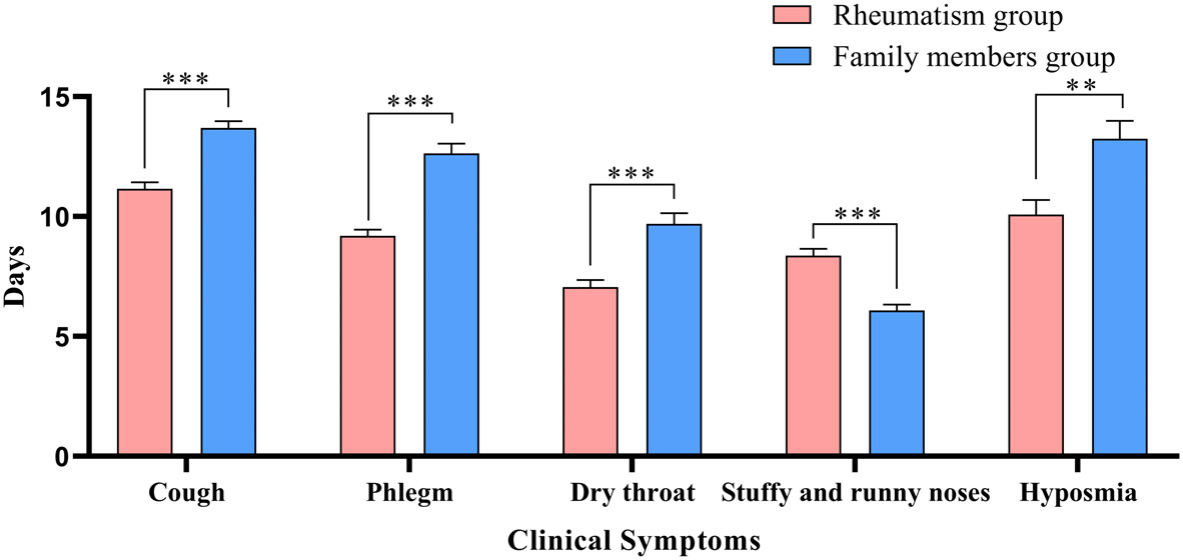
Differences in respiratory symptoms between the two groups. **: *P*<0.01;***: *P*<0.001.

**Figure 2:**
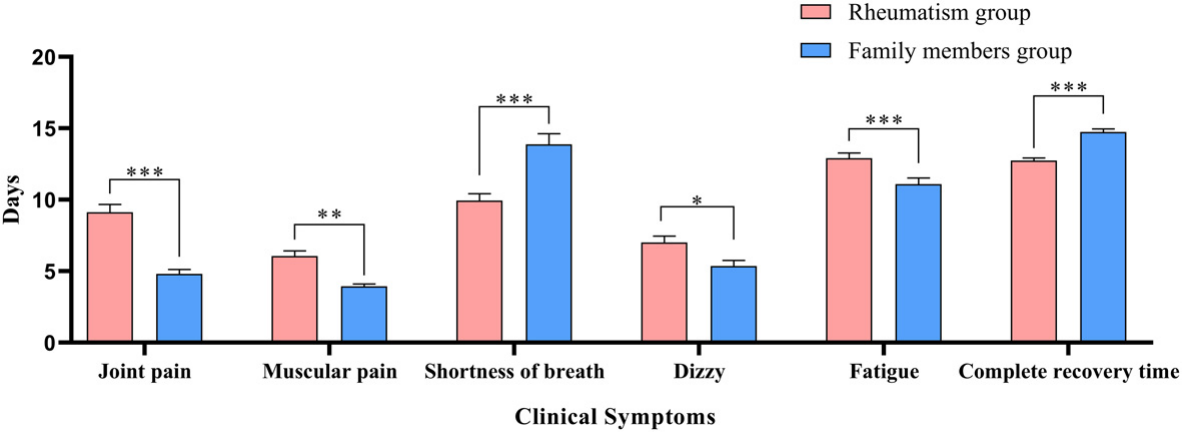
Differences in duration of symptoms between the two groups. *: *P*<0.05;**: *P*<0.01;***: *P*<0.001.

**Table 2** describes the clinical symptoms and characteristics of patients with rheumatic diseases and their family members after infection with COVID-19. There were 958 cases of fever in rheumatism group, 962 cases in family members group, and the difference between the two groups was statistically significant (*P* < 0.05). The number of patients with moderate or high-grade fever in rheumatism group was lower than that in family members group (*P* < 0.05). The duration of symptoms of cough, phlegm, dry throat, hypogeusia, hyposmia and shortness of breath in the rheumatism group was less than that in the family members group, and the difference between the two groups was statistically significant (*P* < 0.05). In addition, the complete recovery time from COVID-19 of patients with rheumatic diseases was 13 days (8.00, 18.00), which was lower than that of family members (16 days) (11.00, 20.00), with statistical difference (*P*<0.001). We mapped indicators of the difference in symptom duration between the two groups (**Figures 1**, **2**).

**Table 2:** Characteristics of Clinical Symptoms of 2,889 Investigated Subjects.

Variables	Rheumatism group	Family members group	*P*
Fever, n(%)	958(62.6)	962(70.9)	<0.001
Low-grade fever	411(26.9)	293(21.6)	0.001
Moderate-grade fever	510(33.3)	559(41.1)	<0.001
High-grade fever	124(8.1)	182(13.4)	<0.001
Duration of high-grade fever (days)	1.50(1.00, 2.38)	2.00(1.00, 2.13)	0.062
Duration of moderate-grade fever (days)	2.00(1.00, 3.00)	2.00(1.00, 3.00)	0.259
Duration of low-grade fever (days)	2.00(1.00, 3.00)	2.00(1.00, 2.00)	0.146
Cough, n(%)	928(60.7)	775(57.0)	0.111
Duration of cough (days)	10.00(5.00, 16.00)	14.00(7.00, 18.00)	<0.001
Phlegm, n(%)	644(42.1)	459(33.8)	<0.001
Duration of phlegm (days)	7.00(5.00, 14.00)	10.00(7.00, 17.00)	<0.001
Sore throat, n(%)	519(33.9)	486(35.8)	0.309
Duration of sore throat (days)	4.00(3.00, 7.00)	4.00(3.00, 7.00)	0.399
Dry throat, n(%)	382(25.0)	300(22.1)	0.072
Duration of dry throat (days)	5.00(3.00, 10.00)	7.00(4.00, 15.00)	<0.001
Itchy throat, n(%)	269(17.6)	195(14.3)	0.020
Duration of itchy throat (days)	7.00(3.00, 15.00)	6.00(3.00, 10.00)	0.079
Stuffy and runny noses, n(%)	508(33.2)	332(24.4)	<0.001
Duration of stuffy and runny noses (days)	7.00(3.00, 10.00)	5.00(3.00, 7.00)	<0.001
Hypogeusia, n(%)	179(11.7)	195(14.3)	0.034
Duration of hypogeusia (days)	10.00(5.00, 14.00)	9.00(5.00, 15.00)	0.288
Hyposmia, n(%)	130(8.5)	143(10.5)	0.063
Duration of hyposmia (days)	10.00(5.00, 14.00)	14.00(7.00, 18.00)	0.002
Joint pain, n(%)	352(23.0)	244(18.0)	0.001
Duration of joint pain (days)	5.00(3.00, 10.75)	3.00(2.00, 6.75)	<0.001
Muscular pain, n(%)	393(25.7)	366(26.9)	0.472
Duration of muscular pain (days)	3.00(2.00, 7.00)	3.00(2.00, 5.00)	0.003
Chest stuffiness, n(%)	241(15.8)	143(10.5)	<0.001
Duration of chest stuffiness (days)	7.00(3.00, 10.00)	7.00(3.00, 12.00)	0.637
Shortness of breath, n(%)	242(15.8)	153(11.3)	<0.001
Duration of shortness of breath (days)	9.00(5.00, 16.00)	12.00(7.00, 20.00)	<0.001
Breathing difficulties, n(%)	84(5.5)	81(6.0)	0.630
Duration of breathing difficulties (days)	7.00(3.00, 10.00)	5.00(3.00, 8.00)	0.070
Headache, n(%)	439(26.7)	305(22.4)	<0.001
Duration of headache (days)	3.00(2.00, 5.00)	3.00(2.00, 5.00)	0.716
Dizzy, n(%)	266(17.4)	180(13.2)	0.002
Duration of dizzy (days)	4.00(2.00, 10.00)	3.00(2.00, 7.00)	0.032
Fatigue, n(%)	572(37.4)	432(31.8)	0.002
Duration of fatigue (days)	10.00(7.00, 18.00)	9.00(5.00, 15.00)	<0.001
Stomachache, n(%)	49(3.2)	28(2.1)	0.064
Duration of stomachache (days)	3.00(2.00, 8.50)	3.00(2.00, 5.75)	0.801
Diarrhea, n(%)	169(11.0)	75(5.5)	<0.001
Duration of diarrhea (days)	3.00(1.75, 5.00)	2.00(2.00, 3.00)	0.226
Nausea, n(%)	158(10.3)	86(6.3)	<0.001
Duration of nausea (days)	4.00(2.00, 7.00)	3.00(2.00, 7.00)	0.532
Emesis, n(%)	111(7.3)	56(4.1)	<0.001
Duration of emesis (days)	2.00(1.00, 3.00)	2.00(1.00, 3.00)	0.745
Palpitation, n(%)	121(7.9)	74(5.4)	0.09
Duration of palpitation (days)	7.00(3.00, 15.00)	7.00(3.00, 15.00)	0.543
Insomnia, n(%)	247(16.1)	113(8.3)	<0.001
Duration of insomnia (days)	10.00(5.00, 20.00)	7.00(4.00, 15.00)	0.058
Inattention phenomenon, n(%)	97(6.3)	76(5.6)	0.432
Duration of inattention phenomenon (days)	10.00(5.00, 20.00)	10.00(5.00, 20.00)	0.704
Decreased execution ability, n(%)	80(5.2)	78(5.7)	0.567
Duration of decreased execution ability (days)	10.00(7.00, 18.50)	10.00(5.00, 20.00)	0.936
Complete recovery time from COVID-19	13.00(8.00, 18.00)	16.00(11.00, 20.00)	<0.001

### 3.3 Factors affecting the complete recovery time from COVID-19

With the complete recovery time from COVID-19 as the outcome, multiple linear regression was used to investigate the effects of related factors (whether suffering from rheumatic diseases, and medication usage during infection, such as NSAIDs, DMARDs, glucocorticoids, biologics, Chinese patent medicine, Chinese herbal decoctions) on the complete recovery time from COVID-19. After controlling for all other variables, suffering from rheumatism and mild rheumatism had a significant negative correlation with the complete recovery time from COVID-19. Compared to those without rheumatic diseases, patients with rheumatic diseases had an average of 2.241 fewer days of the complete recovery time from COVID-19 (*P* < 0.001), among which the complete recovery time from COVID-19 of patients with mild rheumatism was reduced by 4.178 days on average (*P* < 0.001), and the complete recovery time from COVID-19 of patients with severe rheumatism was not significantly different from that of their family members (*P*=0.1672). The use of NSAIDS, glucocorticoids, DMARDs, biologics, Chinese patent medicine, Chinese herbal decoctions during the infection period had a significant negative correlation with the complete recovery time from COVID-19 symptoms, and the average reduction was 1.196 days, 1.669 days, 3.817 days, 2.755 days, 0.677 days and 3.228 days, respectively (*P* < 0.05) (**Table 3**).

**Table 3:** Multiple linear regression analysis affecting the complete recovery time from COVID-19.

Variables	Estimated value (coefficient)	SE	*t*	*P*	95%CI
Suffering from rheumatism	-2.241	0.2463	-9.0977	< 0.001	(-2.724, -1.758)
Suffering from mild rheumatism	-4.178	0.3014	-13.8635	< 0.001	(-4.769, -3.587)
Suffering from severe rheumatism	-0.430	0.3115	-1.3818	0.1672	(-1.041, 0.180)
Use of NSAIDs during infection	-1.196	0.2091	-5.7193	< 0.001	(-1.606, -0.786)
Use of glucocorticoids during infection	-1.669	0.3446	-4.8430	< 0.001	(-2.344, -0.993)
Use of DMARDs during infection	-3.817	0.5676	-6.7245	< 0.001	(-4.93, -2.704)
Use of biologics during infection	-2.755	0.6288	-4.3808	< 0.001	(-3.987,-1.522)
Use of Chinese patent medicine during infection	-0.677	0.2121	-3.1928	0.0014	(-1.093, -0.261)
Use of Chinese herbal decoctions during infection	-3.228	0.5533	-5.8345	< 0.001	(-4.313, -2.143)

### 3.4 Factors influencing the clinical types of COVID-19 infection

With the clinical types of COVID-19 infection (asymptomatic, mild, moderate to severe) as the outcome, multiple logistic regression was used to investigate the effects of related factors (whether suffering from rheumatic diseases, and medication usage during infection, such as NSAIDs, DMARDs, glucocorticoids, biologics, Chinese patent medicine, Chinese herbal decoctions) on the clinical types of COVID-19. Taking asymptomatic type as the reference group, the probability of mild type in patients with rheumatic diseases was 1.278 times that of their family members when other variables were controlled. There might be a positive correlation between the occurrence of mild type and suffering from rheumatism, but there was no statistical difference (*P*=0.0882). The risk of developing moderate to severe type in patients with rheumatic diseases was 0.511 times that of the normal population, which is significantly lower than the risk of moderate to severe type in family members (*P*=0.0026). Compared to those who did not use the corresponding medication, patients who used NSAIDs before infection had a 0.595 times risk of developing mild infections (*P* = 0.0003), and the probability of developing mild infections increased in patients who used glucocorticoids and Chinese herbal decoctions before infection (OR=1.537, 1.773, *P* < 0.05). The probability of developing moderate to severe infections in patients who received DMARDs before infection was 0.350 times that of patients who did not receive the drugs, indicating a significant reduction in the risk of developing moderate to severe infections (*P*<0.001) (**Table 4**).

**Table 4:** Multiple logistic regression analysis affecting clinical types of COVID-19 infection.

Clinical Types of COVID-19	Variables	OR	*P*	95%CI
Mild	Suffering from rheumatism	1.278	0.0882	(0.964, 1.693)
	Use of NSAIDs before infection	0.595	0.0003	(0.449, 0.789)
	Use of glucocorticoids before infection	1.537	0.0090	(1.113, 2.121)
	Use of DMARDs before infection	1.137	0.3311	(0.878, 1.472)
	Use of biologics before infection	1.351	0.0762	(0.969, 1.883)
	Use of Chinese patent medicine before infection	1.453	0.1081	(0.921, 2.293)
	Use of Chinese herbal decoctions before infection	1.773	0.0018	(1.237, 2.54)
Moderate to severe	Suffering from rheumatism	0.511	0.0026	(0.330, 0.791)
	Use of NSAIDs before infection	0.735	0.1687	(0.474, 1.14)
	Use of glucocorticoids before infection	0.709	0.2041	(0.417, 1.206)
	Use of DMARDs before infection	0.350	<0.001	(0.222, 0.550)
	Use of biologics before infection	0.707	0.2226	(0.405, 1.234)
	Use of Chinese patent medicine before infection	0.957	0.9030	(0.468, 1.955)
	Use of Chinese herbal decoctions before infection	1.185	0.5317	(0.696, 2.016)

## 4 Discussion

Rheumatism is an autoimmune disease. Respiratory viruses might increase the incidence of rheumatic diseases represented by rheumatoid arthritis (RA), and they are risk factors for RA (20). Patients with rheumatic diseases often receive long-term treatment with steroids, immunosuppressants or biologics, and are immunosuppressed. Compared with the general population, patients with rheumatic diseases have an increased risk of infection with COVID-19 (10, 11). Infection with COVID-19 may increase disease activity in patients with rheumatoid arthritis, making their symptoms last longer than those without rheumatism (21). However, in the clinical treatment of COVID-19, some drugs used in the treatment of rheumatism (including NSAIDs, glucocorticoids, DMARDs and biologics, etc.) are also used in the treatment of COVID-19 (21). Are respiratory symptoms worse in patients with rheumatic diseases infected with COVID-19? Could commonly used drugs (including NSAIDs, glucocorticoids, DMARDs and biologics) in patients with rheumatic diseases affect the prognosis of COVID-19? Those are controversial.

This study conducted a retrospective investigative analysis of the clinical manifestations of COVID-19 infection among patients with rheumatic diseases and their family members. The study found that most patients and their families had fever, cough, phlegm and other symptoms. However, the duration of cough, phlegm, dry throat, hyposmia and shortness of breath in patients with rheumatic disease was shorter than that of the family members group. Furthermore, the complete recovery time of patients with rheumatic disease from COVID-19 was significantly shorter than that of the family members group. Previous studies have also shown that the symptoms of patients with rheumatic diseases infected with the COVID-19 are similar to those of the general population, with cough and fever as the main common symptoms (22). The results of this study showed that most patients with rheumatic diseases infected with COVID-19 have mild symptoms, with fever predominantly being low-grade or moderate. The clinical symptoms of patients with rheumatic disease after infection with COVID-19 were relatively milder, which might be related to the use of glucocorticoids, Chinese herbal decoctions, and other drugs before infection. At the same time, the use of DMARDs before infection was associated with a lower probability of developing severe cases in COVID-19 patients. However, patients who used NSAIDs before infection had a lower risk of developing mild COVID-19. Previous studies have also indicated that long-term use of NSAIDs by patients with rheumatic diseases may cover up fever and other symptoms (5), which could delay the treatment of COVID-19 and make the condition worse. A randomized controlled trial has shown that early inhalation of budesonide in patients with COVID-19 may reduce hospitalization or death rates in patients with reduced recovery time (23). Another randomized controlled trial found that COVID-19 patients who received treatment with Tixagevimab and Cilgavimab early in their infection had lower rates of severe case and mortality than those in the placebo group (24). However, the use of biologics before COVID-19 infection in patients in this study did not lead to a higher rate of mild COVID-19. Also, patients who used biologics after infection had a shorter recovery time.

This study found that the complete recovery time from COVID-19 was shorter in patients with rheumatic diseases than in their family members, mainly in patients with mild rheumatism, while not in patients with severe rheumatism. Therefore, it is known that actively taking drugs to treat rheumatism on time and controlling the rheumatism can help the recovery of the COVID-19. This study found that the use of NSAIDs, glucocorticoids, DMARDs, biologics, Chinese patent medicine, and Chinese herbal decoctions during infection significantly shortened the complete recovery time from COVID-19. Patients using DMARDs, Chinese herbal decoctions and biologics had shorter complete recovery time from COVID-19, with a reduction of 3.817, 3.228, and 2.755 days, respectively, which might be related to the fact that these drugs could improve the condition of COVID-19 in addition to treating rheumatism. Braun-Moscovici et al. found that the immune-modulating effects of anti-rheumatic drugs made them capable of preventing or treating viral infections in patients with rheumatic diseases when infected with COVID-19 (25). During the outbreak of COVID-19, some DMARDs were recommended for the treatment of COVID-19, and the results of a small randomized controlled trial showed that the level of C-reactive protein (CRP) in 5 COVID-19 patients was significantly lower after receiving treatment with leflunomide, and inflammation was controlled, thus preliminarily confirming the efficacy of leflunomide in treating COVID-19 (26). Methotrexate, a representative drug for RA, can effectively resist the cytokine storm of COVID-19 (27). Biologics used for RA treatment have also been applied to the treatment of COVID-19, such as tocilizumab, which has shown good therapeutic effects in COVID-19 patients (28). Traditional Chinese medicine has played an important role in the prevention and treatment of COVID-19 (29, 30). *Diagnosis and Treatment of COVID-19 (10th edition)* published by the National Health Commission of the People’s Republic of China (3) also recommends a variety of Chinese medicines for the treatment of COVID-19, such as Lianhua Qingwen capsules, which can inhibit SARS-CoV-2 replication and inhibit the production of pro-inflammatory cytokines at the mRNA level (31). The results of an international multi-center RCT conducted in 2019 also showed that Lianhua Qingwen capsules can accelerate symptom resolution and promote recovery of mild to moderate COVID-19 patients (32). A meta-analysis showed that compared with the use of modern medicine alone, the combination of Lianhua Qingwen capsule and modern medicine in COVID-19 patients could better improve clinical symptoms such as cough, fatigue, chest tightness, shortness of breath, and shorten the duration of fever (33). A randomized controlled trial showed that compared with the conventional treatment alone, the 29 COVID-19 patients who received Xuebijing injection treatment in addition to the conventional treatment had shorter duration of fever, cough, chest tightness and shortness of breath and a better clinical outcome (34). Some of the ingredients in TCM used to treat rheumatoid arthritis also overlap with those in drugs for treating COVID-19. For example, Wangbi granule is a representative TCM for treating rheumatoid arthritis (35). It can improve symptoms and synovitis associated with rheumatism, as well as reduce levels of serum inflammatory cytokines/chemokines (36), and Fangfeng (*Saposhnikovia divaricate*), Duhuo(*Heracleum hemsleyanum*) and Guizhi(*Ramulus Cinnamomi*) have anti-inflammatory and antiviral effects, and are recommended for use in the treatment of COVID-19 (37–39) according to *Diagnosis and Treatment of COVID-19 (10th edition)* published by the National Health Commission of the People’s Republic of China (3).

There are also some limitations in this study. The COVID-19 infected patients in this survey were mainly patients in the rheumatology and immunology outpatient department or inpatient department and their families, which will undoubtedly make it difficult for patients who cannot see a doctor to participate in this study. Since critical cases of COVID-19 are generally hospitalized in ICU, it is difficult to collect data on this type of patients, which may cause bias in the study results. Secondly, this study only included patients with COVID-19 in China, which may result in racial bias. Finally, due to the multiple drugs taken by patients (including NSAIDs, glucocorticoids, DMARDs, biologics, Chinese patent medicine, and Chinese herbal decoctions), each type includes a variety of drugs, it is impossible to further analyze the effects of specific drugs on clinical symptoms and prognosis of patients. As many patients were not able to accurately tell the specific names of the biologics used, this study was unable to analyze which specific biologics would affect the outcomes of COVID-19 infection.

## 5 Conclusion

This study found that patients with rheumatic diseases had a higher incidence of mild cases with COVID-19 and a significantly lower risk of developing moderate to severe cases compared to their family members, which was related to the use of glucocorticoids, DMARDs and Chinese herbal decoctions before COVID-19 infection. In addition, patients with mild rheumatism have a shorter time to complete recovery after infection with COVID-19, which is associated with the use of NSAIDs, glucocorticoids, DMARDs, biologics, Chinese patent medicine, and Chinese herbal decoctions during the infection period.

## Data Availability

The original contributions presented in the study are included in the article/supplementary material. Further inquiries can be directed to the corresponding authors.
